# Comparative analysis of clinical efficacy between laparoscopic and open pancreaticoduodenectomy

**DOI:** 10.1097/MD.0000000000033588

**Published:** 2023-04-21

**Authors:** Linyang Li, Zhang Bo, Qiuhua Liu, Gang Wang, Wangji Zhang, Qinyu Liang

**Affiliations:** a The Affiliated Zhangjiagang Hospital of Soochow University, Suzhou City, China.

**Keywords:** laparoscopy, open, pancreaticoduodenectomy, prognosis

## Abstract

Laparoscopic pancreaticoduodenectomy (LPD) is a technically demanding procedure but is gradually gaining acceptance in clinical practice. This study was performed to compare the short-term outcomes of LPD with open pancreaticoduodenectomy (OPD). The perioperative data of the patients who underwent LPD (n = 25) and OPD (n = 40) from January 1, 2017 to December 31, 2021 at Zhangjiagang Hospital Affiliated to Soochow University were collected and retrospectively analyzed. All patients received R0 resection, and none of the patients died within the perioperative period. The preoperative data (gender, age, body mass index [BMI], and preoperative bilirubin), the intraoperative data (operative time, number of retrieved lymph nodes), and postoperative data (level 1 monitoring time, postoperative fluid diet time, postoperative fluid feeding time, and hospitalization cost) were comparable between the 2 groups (*P* > .05). The estimated blood loss, abdominal drainage tube removal time, postoperative hospital stay, catheter removal time, and analgesic drug use were significantly lesser in the LPD group, when compared to the OPD group (*P* < .05). LPD is safe and feasible. Compared to OPD, LPD has less surgical trauma, less intraoperative bleeding, and faster postoperative recovery.

## 1. Introduction

Pancreatic cancer is the seventh leading cause of cancer death. In 2020, there were about 466,000 deaths, accounting for about 4.7 percent of deaths from malignant tumors. About 47.1% of new cases and 48.1% of deaths globally occur in Asia.^[1]^ For the past few decades, pancreaticoduodenectomy (PD) has been the primary treatment for pancreatic cancer.

Laparoscopic pancreaticoduodenectomy (LPD) is a technically difficult minimally invasive procedure, since the pancreatic head and duodenum are located in the retroperitoneum, and lie in close proximity to the major vessels. However, due to technological innovations and improvements in surgical techniques, LPD has gained increasing popularity and acceptance at several surgical centers worldwide.^[2]^ Since Gagner and Pomp reported their first LPD experience in 1994,^[3]^ LPD has gradually been accepted in clinical practice, and clinical studies that compared LPD and open pancreaticoduodenectomy (OPD) have been carried out at major medical centers in China and abroad. Previous studies have reported that LPD can be a promising alternative to OPD in selected patients, with good surgical and oncology outcomes.^[4–6]^ However, some researchers consider that although LPD is safe and feasible, the overall complications and perioperative mortality are comparable to OPD. Hence, there is much debate on the merits and demerits of LPD. We present a case series that compared the short-term outcomes of the LPD and OPD performed at our center.

## 2. Materials and methods

### 2.1. Clinical data

The data of patients, who underwent PD at Zhangjiagang Hospital Affiliated to Soochow University from January 1, 2017 to December 31, 2021, were retrospectively analyzed. According to the surgical technique, these patients were divided into 2 groups: LPD group and OPD group. All patients having periampullary tumors (including the ampulla itself, lower segment of common bile duct, duodenal papilla, and pancreatic head) without distant metastasis, and no other serious organ insufficiency of the heart, lungs, brain, kidneys, or other important organs were included. Patients with pancreatic head tumor diameter >4 cm were included in the OPD group. All surgeries were performed by surgeons with experience in open PD and minimally invasive surgery. Experience in LPD was in the early phase of the learning curve due to the small number of cases. In this early phase of the learning curve, the authors selected young patients (age <50 years, good cardiopulmonary function, body mass index [BMI] < 28) with good general condition having tumors in the lower segment of common bile duct or periampullary region and dilated common bile duct and pancreatic duct without vascular or pancreatic invasion and no history of complicated abdominal surgery for LPD. All patients provided written informed consent for the operation.

The reviewed data included the following: age, gender, BMI, surgical and postoperative recovery indicators, postoperative complications, postoperative pathological parameters, length of hospital stay, and cost of treatment. The surgical indicators included operative time and intraoperative blood loss, and the postoperative recovery indicators included postoperative intensive care unit (ICU) stay, total duration of drainage tube, postoperative urinary tube removal time, and postoperative fluid feeding time. The postoperative complications were classified as postoperative pancreatic fistula, biliary leakage, abdominal bleeding, delayed gastric emptying, ascites, and perioperative death. The postoperative pathological parameters were the R0 resection rate and number of dissected lymph nodes.

The diagnosis of postoperative pancreatic leakage was based on the criteria formulated by the International Organization of Pancreatic Surgery in 2016.^[7]^ Other complications were graded according to the Clavien-Dindo classification system (higher than grade III was defined as a major complication).^[8]^ Delayed gastric emptying were defined according to the established international consensus.^[7]^

### 2.2. Ethics

The present study was approved by the Ethics Committee of Zhangjiagang Hospital Affiliated to Soochow University (number:ZJGYYLL-2022-07-010). Written informed consent was obtained from each participant. The study was conducted in accordance to the Helsinki Declaration.

### 2.3. Operative methods

#### 2.3..1. Preoperative treatment.

All patients were routinely examined by contrast-enhanced computed tomography or magnetic resonance imaging before surgery, gastroduodenoscopy, contrast-enhanced ultrasound, and other examinations, as required. Patients with significantly elevated bilirubin (≥340 μmol/L) before surgery were treated with preoperative biliary drainage. All patients were selected for laparoscopic or open surgery, based on the informed consent of the patient and their families.

#### 2.3..2. Laparoscopic pancreaticoduodenectomy.

Pneumoperitoneum was established using the Veress needle or open Hasson technique, and a 10 mm port was placed below the umbilicus. Then, a 12 mm port was placed in the subcostal region in the left axillary line, and a 5 mm port was placed in the midclavicular line on the left and right sides. Classical PD was performed, which included the distal stomach, common bile duct, uncinate process of the pancreas, horizontal part of the duodenum, and surrounding lymph nodes. The gallbladder was temporarily retained for traction during the operation. A 3 cm incision was made below the umbilicus to remove the specimen.

Reconstruction method: The choice of reconstruction was duct-to-mucosa pancreatojejunostomy. A supporting tube was placed in the pancreatic duct, and the posterior wall of the pancreas was continuously sutured to the jejunum using a 3-0 prolene suture. After opening the jejunum at an appropriate position, the supporting tube was placed in the jejunal opening, and the pancreatic duct and small intestine were sutured with 5-0 prolene using 3 stitches. The cut margin of the pancreas was sutured to the jejunum using 3-0 prolene, and this was continuously stitched posteriorly and anteriorly. Approximately 5 cm away from the pancreaticojejunal anastomosis, a jejunal opening of 1 cm was made. The cut end of the common bile duct was continuously anastomosed to the jejunum in an end-to-side fashion using 4-0 prolene sutures. Then, the gall bladder was removed, and the transverse mesocolic opening was closed. Next, the small intestine and stomach were anastomosed at 40 cm from the mesangial foramen. Initially, gastrojejunal anastomosis was performed using a long articulating endoscopic linear cutter. Then, the common opening was closed by intermittent sutures.

#### 2.3..3. Open pancreaticoduodenectomy.

The abdomen was opened in layers, and the peritoneal cavity was explored for metastasis. The second part of the duodenum and the head of the pancreas were fully mobilized using Kocher technique. Then, cholecystectomy was performed, and the common bile duct was divided just below the hepatic hilum. Afterwards, the distal end of the stomach was divided using a cutting stapler. Next, the pancreas was cut above the superior mesenteric vein. Then, the uncinate process and head of the pancreas were separated, and the surrounding lymph nodes were dissected. Afterwards, the jejunum was divided at approximately 10 cm from the ligament of Treitz.

Reconstruction method: A 10F thin tube was inserted into the pancreatic duct. Then, duct-to-mucosa pancreaticojejunostomy was performed in single layer using non-absorbable sutures. Afterwards, end-to-side hepaticojejunal anastomosis was performed using the 1-layer method at approximately 10 cm away from the pancreaticojejunostomy using 5-0 prolene sutures. Subsequently, end-to-side manual gastrojejunostomy was performed at approximately 45 cm away from the pancreaticojejunostomy using a long articulating endoscopic linear cutter.

### 2.4. Postoperative management

In the early postoperative period, the patients were kept fasting. Medications included proton pump inhibitors, intravenous antibiotics, analgesics, fluids and liver protection drugs. Blood routine and biochemical tests were conducted regularly. Patients were started on enteral nutrition through jejunostomy tube on third or fourth postoperative day. Color and volume of the drainage fluid were observed, and amylase content of drainage fluid was detected. The timing of drainage tube removal was determined by the color of the drainage fluid, drain output and postoperative abdominal computed tomography findings.

### 2.5. Statistical analysis

SPSS version 25.0 was used to analyze the perioperative data of patients included in the present study. The quantitative data were expressed as mean ± standard deviation, and compared using *t* test. The qualitative data were compared using *χ*^2^-test. *P* < .05 was considered statistically significant.

## 3. Results

The present study included 25 and 40 patients in the LPD and OPD groups, respectively. R0 resection was achieved for all patients. None of these patients required conversion from LPD to OPD. The preoperative characteristics of patients in the 2 groups are presented in Table 1. The preoperative data (gender, age, BMI) of patients in the 2 groups were similar (*P* > .05). There was no significant difference in tumor size, American Society of Anesthesiologists grade, number of diabetes mellitus patients, number of preoperative biliary drainage and preoperative bilirubin between the 2 groups.

**Table 1:** Patient demographic and preoperative clinical characteristics.

Items	LPD (25)	OPD (40)	*P*
Male gender	17	23	NS
Age (yr)	63.88 ± 9.20	67.88 ± 7.19	NS
BMI (kg/m^2^)	22.86 ± 2.45	22.40 ± 2.86	NS
PB (μmol/L)	89.06 ± 98.86	105.16 ± 109.86	NS
Tumor size (cm)	2.76 ± 0.88	3.01 ± 1.33	NS
ASA			NS
II	13	23	
III	12	16	
DM	4	9	NS
PBD	4	6	NS

ASA = American Society of Anesthesiologists grade, BMI = body mass index in kg/m^2^, DM = diabetes mellitus, LPD = laparoscopic pancreaticoduodenectomy, NS = no significant difference, OPD = open pancreaticoduodenectomy, PB = Preoperative bilirubin, PBD = Preoperative biliary drainage.

The intraoperative and postoperative parameters of these patients are presented in Table 2. The number of removed lymph nodes in the 2 groups were similar. The intraoperative blood loss was significantly lesser and the operative time was significantly longer in the LPD group, when compared to the OPD group (*P* < .05). In terms of postoperative recovery, the duration of ICU stays, and duration of postoperative liquid diet of patients in the 2 groups were similar (*P* > .05). The abdominal drainage tube removal time in LPD group was less than that in OPD group (*P* < .05). The urinary catheter removal time and number of analgesic drugs used were lesser in the LPD group, when compared to the OPD group (*P* < .05). None of the patients died during the perioperative period.

**Table 2:** Perioperative parameters.

Items	LPD (25)	OPD (40)	*P*
OT (min)	338.60 ± 79.23	269.42 ± 68.67	<.05
OBL (mL)	248.00 ± 207.40	460.00 ± 390.13	<.05
NLNR	11.64 ± 6.38	10.23 ± 6.33	NS
R0 resection	25 (100%)	40 (100%)	NS
Intensive care time (d)	19.60 ± 7.55	20.70 ± 10.99	NS
All drainage tube removal time (d)	11.80 ± 4.50	15.72 ± 7.73	<.05
Urine catheter removal time (d)	1.76 ± 0.88	2.75 ± 1.68	<.05
Postoperative fluid diet time (d)	8.00 ± 5.24	7.58 ± 4.22	NS
NADU	1.12 ± 1.62	4.45 ± 6.55	<.05
Morbidity (CD ≥ II)	8	13	NS
Pancreatic fistula	4	7	NS
DGE	3	4	NS
Hemorrhage Bile leakage	1 1	2 1	NS NS

CD = Clavien-Dindo classification, DGE = delayed gastric emptying, LPD = laparoscopic pancreaticoduodenectomy, NADU = number of analgesic drugs used, NLNR = number of lymph nodes resected, OBL = operative blood loss, OPD = open pancreaticoduodenectomy, OT = operation time.

The postoperative complications are presented in Table 2. There were 9 cases in the LPD group, with a complication rate of 36% (9/25). Among these 9 cases, there were 4 cases of pancreatic leakage (all grade B pancreatic leakage), which were treated with abdominal irrigation, fluid rehydration and antimicrobials. One case of abdominal bleeding was treated with embolization of the splenic artery by interventional angiography, and 3 case of delayed gastric emptying (grade 2)was treated by fasting, nasogastric decompression, nutritional support, and gastrointestinal motility drugs. There was 1 case of bile leakage managed conservatively. Furthermore, fifteen patients in the OPD group developed complications, and the complication rate was 37.5% (15/40). Major complications (CD grade ≥ II) occurred in both groups (LPD, 8 vs OPD,14) had no significant difference. There were 8 patients with pancreatic leakage, and all cases were grade B pancreatic leakage. Furthermore, 4 patients had delayed gastric emptying (grade 2). These patients improved after fasting, nasogastric decompression, nutritional support, and gastrointestinal motility stimulants. Moreover, 2 patients with abdominal bleeding were treated with reoperation and blood transfusion. There was 1 case of bile leakage treated conservatively.

The hospitalization indicators are presented in Table 3. The duration of postoperative hospitalization days was significantly lesser in the LPD group, when compared to the OPD group (*P* < .05). However, the hospitalization expenses between these 2 groups were similar (*P* > .05).

**Table 3:** Hospitalization indicators.

Items	LPD	OPD	*P*
DPHD (d)	21.20 ± 6.97	26.38 ± 11.08	<.05
Hospital costs (10K RMB)	9.27 ± 2.48	9.56 ± 3.50	NS

DPHD = duration of postoperative hospitalization days, LPD = laparoscopic pancreaticoduodenectomy, OPD = open pancreaticoduodenectomy.

## 4. Discussion

Undeniably, the concept of precise and minimally invasive surgery has gradually gained popularity among people. LPD has the advantages of small wound, less intraoperative blood loss, more precise operation, less postoperative pain, and short recovery time.^[9]^ At present, a number of studies have confirmed^[3,4,10,11]^ that LPD has advantages over OPD, in terms of reducing surgical bleeding, relieving postoperative pain, and shortening the postoperative recovery time. The results of the present study confirmed the benefits of LPD over OPD. It was considered that with the continuous development of laparoscopic technology and improvements in surgical expertise, the advantages of laparoscopic technology would be further reflected.

A number of studies^[2,12]^ have reported that LPD requires a longer operation time, which means prolonged anesthesia and pneumoperitoneum, thereby increasing the risk of perioperative cardiopulmonary complications. Similar results were found in the present study. However, the operative time of LPD at our center gradually decreased from 460 minutes in the initial period to 300 minutes in the later period of our surgical experience. In the LPD group, the operative time of the first case was the longest due to the lack of experience and limited skills of our surgical team in doing pancreatojejunostomy and hepaticojejunostomy laparoscopically. However, with the increase in the number of surgical cases, the laparoscopic skills improved, especially the time to perform laparoscopic pancreaticojejunostomy and biliojejunostomy leading to gradual shortening of the operation time. Inorder to reduce the operation time, we also performed gastrojejunal anastomosis through the small epigastric incision made to retrieve the specimen. However, it did not shorten the operation time significantly. Hence, these findings indicate that LPD has a steep learning curve, and that the operative time gradually shortens with the increase in experience of the surgical team.

Cost is another obstacle in the popularization of LPD. High cost associated with the use of laparoscopic instruments and staplers may make it difficult for patients to accept LPD. However, in the present study results, there was no statistical difference in hospitalization cost between LPD and OPD. In addition, Gerber et al^[13]^ reported that the cost of LPD and OPD are similar, while the total nursing cost was even lower than OPD. This means that LPD may be more acceptable, in terms of total cost, when compared to OPD, in the future.

In terms of surgical resection, both OPD and LPD achieved R0 resection, and the number of removed lymph nodes in both groups were similar, suggesting that LPD was not inferior to OPD in achieving R0 resection and adequate lymphadenectomy. These findings are consistent with that of existing studies.

PD is associated with a high incidence of postoperative complications. A meta-analysis conducted by Professor Boggi^[10]^ revealed that the total incidence of postoperative complications after LPD was 41.2% (252/611). Furthermore, a multi-center retrospective study conducted in China reported postoperative complications in almost half of LPD patients.^[11]^ In the present study, postoperative complications occurred in 36% and 37.5% of cases after LPD and OPD, respectively, which were lower than those previously reported. However, LPD has no advantage over OPD in reducing complications.

One of the major complications of PD is anastomotic leakage, especially pancreaticoenteric anastomosis.^[14]^ Pancreaticojejunostomy is the most preferred anastomotic technique for the pancreatic stump. In the present study, there were 4 cases of postoperative pancreatic leakage (16%) in the LPD group and 8 cases of postoperative pancreatic leakage (20%) in the OPD group. In our hospital, end-to-side pancreaticojejunostomy anastomosis has always been used for LPD. A meta-analysis conducted by Hua J^[15]^ revealed that catheter-to-mucosal pancreaticojejunostomy had no advantage in preventing pancreatic leakage, but this could reduce anastomotic bleeding, and may be beneficial for anastomotic healing. Therefore, the investigators intend to perform catheter-to-mucosal pancreaticojejunostomy in future LPDs. In addition, a number of hospitals in China perform pancreatogastrostomy. A domestic meta-analysis^[16]^ suggested that pancreatogastrostomy can produce good results, in terms of pancreatic leakage. Furthermore, Fernandez-Cruz et al^[17]^ reported a lower incidence of postoperative pancreatic fistula in pancreatogastrostomy, when compared to pancreaticojejunostomy, through a randomized controlled trial (4% vs 22%, *P* < .01). However, pancreatogastrostomy requires intraoperative gastric partitioning, which is quite complicated, especially in the case of laparoscopy, and there are higher requirements for the surgeon. Therefore, the author considered that pancreatogastrostomy is still too early to be popularized.

There were some limitations in the present study. First, the present study was a single-center study with a small sample size. Second, merely the short-term outcomes were investigated. Third, the postoperative ICU stay and duration of hospital stay were prolonged in this study due to institutional policy of gradual removal of endotracheal tube, close monitoring in ICU, delayed enteral feeding and prolonged observation in hospital due to safety concerns by the treating physicians and the family members. However, in future we intend to reduce the postoperative hospital stay of the patients by adopting enhanced recovery protocols. Fourth, the steep learning curve of LPD contributed significantly to its long operative time.

Briefly, the present study revealed that LPD has advantages, in terms of lesser intraoperative blood loss and faster postoperative recovery indicators. Furthermore, the operation time was longer, when compared to OPD. However, the operation time gradually decreased with increasing experience. Therefore, with the advancements in the laparoscopic equipment and the standardization of the surgical technique of LPD, the popularity of LPD would be accelerated due to its advantages over OPD.

## Author contributions

**Conceptualization:** Zhang Bo.

**Data curation:** Linyang Li, Zhang Bo.

**Investigation:** Linyang Li.

**Methodology:** Linyang Li, Qiuhua Liu, Gang Wang, Wangji Zhang, Qinyu Liang.

**Project administration:** Zhang Bo.

**Resources:** Linyang Li, Zhang Bo, Qiuhua Liu, Gang Wang, Wangji Zhang, Qinyu Liang.

**Software:** Linyang Li.

**Validation:** Linyang Li, Zhang Bo.

**Visualization:** Linyang Li, Gang Wang, Wangji Zhang, Qinyu Liang.

**Writing – original draft:** Linyang Li.

**Writing – review & editing:** Zhang Bo, Qiuhua Liu.
